# Accessory and Central α-helices of Complexin Selectively Activate Ca^2+^ Triggering of Synaptic Exocytosis

**DOI:** 10.3389/fnmol.2018.00061

**Published:** 2018-02-26

**Authors:** Yi Yu, Su Chen, Xiaoqiang Mo, Jihong Gong, Chenhong Li, Xiaofei Yang

**Affiliations:** ^1^Key Laboratory of Cognitive Science, Hubei Key Laboratory of Medical Information Analysis and Tumor Diagnosis & Treatment, Laboratory of Membrane Ion Channels and Medicine, College of Biomedical Engineering, South-Central University for Nationalities, Wuhan, China; ^2^Youjiang Medical University for Nationalities, Baise, China; ^3^Key Laboratory of Molecular Biophysics of Ministry of Education, College of Life Science and Technology, Huazhong University of Science and Technology, Wuhan, China

**Keywords:** complexin, Ca^2+^ triggered exocytosis, spontaneous release, SNARE protein, synaptic vesicle

## Abstract

Complexins, binding to assembling soluble NSF-attachment protein receptor (SNARE) complexes, activate Ca^2+^ triggered exocytosis and clamp spontaneous release in the presynaptic terminal. Functions of complexin are structural dependent and mechanistically distinct. To further understand the functional/structural dependence of complexin, here we show that the accessory and central α-helices of complexin are sufficient in activation of Ca^2+^ triggered vesicle fusion but not in clamping spontaneous release. Targeting the two α-helices to synaptic vesicle suppresses spontaneous release, thus further emphasizing the importance of curvature membrane localization in clamping function.

## Introduction

Neurotransmitter release is mediated by Ca^2+^ triggered synaptic vesicle fusion. Like most intracellular membrane fusion, synaptic vesicle fusion mediated by soluble NSF-attachment protein receptor (SNARE) and SM (for “Sec1-Munc18 like”) proteins (Rizo and Rosenmund, 2008; Südhof and Rothman, 2009). In presynaptic terminals, syntaxin-1, SNAP-25 and synaptobrevin/VAMP2 (vesicle-associated membrane protein) form a tight complex that forces membranes into close proximity, and Munc18-1 binds to the SNARE complex to catalyze fusion (Südhof and Rothman, 2009). During this process, many other proteins have been involved to promote fusion, including Munc13, synaptotagmin and complexin (Cpx; Geppert et al., 1994; McMahon et al., 1995; Wang et al., 2017). Munc13 is critical in maintaining the readily releasable pool size (Richmond et al., 1999; Varoqueaux et al., 2002) and interacts with Munc18-syntaxin complex to explore the syntaxin linker region in initiation of vesicle fusion (Wang et al., 2017). Synaptotagmin, known as Ca^2+^ sensors, triggers fusion pore opening via the binding of its C2 domains to phospholipids and SNARE complexes (Fernández-Chacón et al., 2001; Wang et al., 2016). And Cpx functionally cooperates with synaptotagmin in regulating synaptic exocytosis (McMahon et al., 1995; Reim et al., 2001; Zhou et al., 2017).

Cpxs are small (~130 residues) and evolutionarily conserved SNARE-binding proteins (Yang et al., 2015). The functions of Cpx in activating Ca^2+^-triggered vesicle release and in clamping spontaneous exocytosis are confirmed not only in *C. elegans* and *Drosophila* (Hobson et al., 2011; Martin et al., 2011; Buhl et al., 2013), but also in mice (Maximov et al., 2009; Yang et al., 2010). Among the four mammalian Cpxs, Cpx1 and Cpx2 are the major expressed isoforms in neurons (McMahon et al., 1995). Knockout both Cpx1 and Cpx2 leads the mice lethal (Reim et al., 2001). Here, we focus on the Cpx1 isoform to briefly summarize most relevant results. Cpx can be divided into four domains: flexible N- and C-terminal domains, an accessory and a central α-helices (Chen et al., 2002). A large number of studies of Cpx function with different approaches has discovered different functions for Cpx in synaptic fusion with distinct sequence requirements. The N-terminal domain (residues 1–26) of Cpx plays a role for fast synchronous Ca^2+^ triggering of exocytosis (Xue et al., 2007; Maximov et al., 2009). The accessory domain (residues 27–47) clamps spontaneous fusion in neurons (Xue et al., 2007; Yang et al., 2010) and it suppresses Ca^2+^-independent fusion *in vitro* systems (Giraudo et al., 2006; Lai et al., 2014; Krishnakumar et al., 2015). The C-terminal domain (residues 71–134) is not only important for both clamping and priming roles (Kaeser-Woo et al., 2012; Dhara et al., 2014; Wragg et al., 2017), but also sensitive to membrane curvature, and it thus localizes Cpx to the synaptic membrane (Wragg et al., 2013; Snead et al., 2014, 2017; Gong et al., 2016). Binding to SNARE complex via the central α-helical domain (residues 48–70; Bracher et al., 2002; Chen et al., 2002) is essential for all functions of Cpx (Maximov et al., 2009; Yang et al., 2013). The differential functional/structural dependence of Cpx strongly argues that these functions are mechanistically distinct.

Although the functions of Cpx and the roles of each domain in the protein have been extensively investigated, the question that what is the minimal functional sequence of Cpx is still unsolved. To address this question, here we investigate all the possible domain combinations of Cpx in Cpx1/2 knockdown (KD) neurons. We found that the central α-helix alone didn’t reverse any phenotype in Cpx deficient mouse neurons, while the accessory and central α-helices together rescued the inactivation of evoked neurotransmitter release, but did not clamp the spontaneous mini release. Moreover, we demonstrated that the vesicular localization helped the two α-helices in suppressing spontaneous fusion. Our results thus suggested that the accessory and central α-helices were the minimal functional structure to activate exocytosis.

## Materials and Methods

### Plasmid Construction

The complexin KD and wild-type complexin (Cpx^WT^) rescue constructs were described previously (Maximov et al., 2009). The mutants contained central α-helix alone (Cpx^48–70^), accessory and central α-helices together (Cpx^27–70^), flanking N-terminal domain to central α-helix (Cpx^1–27 + 48–70^) or accessory and central α-helices fused with cysteine-string protein-α (CSPα; Cpx^27–70-CSPα^) were generated by gene synthesis and were cloned downstream of the human ubiquitin promoter in the L309 lentiviral vector.

### HEK293T Cell Culture

HEK293T cells (CRL-11268, ATCC) were grown in a humidified atmosphere incubator (Thermo) with 5% CO_2_ at 37°C. The culture medium contained Dulbecco’s modified Eagle’s medium (Gibco), 10% fetal bovine serum, and penicillin-streptomycin (50 μg/ml and 50 μg/ml).

### Neuronal Culture

The dissociated cortical neurons were dissected from postnatal day 0 (P0) of WT Kunming mice, dissociated by 0.25% trypsin-EDTA digestion for 12 min at 37°C, plated at 12 mm diameter circular glass coverslips coated with poly-L-lysine (Sigma), and cultured in MEM (GIBCO) supplemented with 2 v/v% B27 (GIBCO), 0.5 w/v% glucose, 100 mg/l transferrin, 5 v/v% fetal bovine serum (GIBCO) and 2 mM Ara-C (Sigma). Wild type mice were fed by mouse facility of South-Central University for Nationalities. No live animals were directly used in this study. All animal procedures were performed in accordance with South-Central University for Nationalities animal use rules and the requisite approvals of animal use committees.

### Lentiviruses Preparation

Lentiviral expression vectors and three helper plasmids (pRSV-REV, pMDLg/pRRE and pVSVG) were co-transfected into HEK293 cells. The transfections were carried out using the polyethylenimine (PEI, 1 mg/ml in ddH_2_O) method with the ratio at PEI:pFUGW:pVSVg:RRE:REV = 24:3:1:2:2. The virus-containing medium was harvested 48 h after transfection and subsequently cleaned with a 3000 *g* centrifuge and a 0.45 μm filtration (Millipore). The virus was then concentrated by a sucrose-containing buffer (50 mM Tris-HCl, pH 7.4, 100 mM NaCl, 0.5 mM ethylene diaminetetraacetic acid [EDTA]) at a 4:1 v/v ratio and centrifuged at 4°C. For re-suspension the virus, Phosphate Buffered Saline (PBS) was added to the tube at the fridge with a cover for recovery overnight. All steps were performed under level II biosafety conditions. Neurons were infected with lentiviruses at days *in vitro* (DIV) 5–6 and analyzed at DIV 13–14.

### Immunocytochemistry

Various lentiviruses infected mouse cortical neurons were fixed in 4% paraformaldehyde and permeabilized with 0.2% Triton X-100, incubated with anti-complexin (polyclonal; L669) and anti-vGlut1 (monoclonal; N28/9 (Neuromab)) primary antibodies in PBS with 5% BSA, washed, and stained with polyclonal anti-complexin and monocolonal anti-vGlut1 and visualized using Alexa Fluor 488 goat anti-rabbit and Alexa Fluor 546 goat anti-mouse secondary antibodies (Molecular Probes). Images were acquired by using a Nikon C2 confocal microscope equipped with a 60× oil-immersion objective. We measured the average pixel intensities by manually tracing each dendrite, with a >2-fold background signal. Identical settings were applied to all samples in each experiment.

### Electrophysiological Recordings

Electrophysiological recordings were performed in whole-cell patch-clamp mode at room temperature using concentric extracellular stimulation electrodes. Patch pipettes were pulled from borosilicate glass capillary tubes (World Precision Instruments, Inc.) by using a P-97 pipette puller. The resistance of pipettes filled with intracellular solution varied between 3 MOhm and 5 MOhm. After formation of the whole-cell configuration and equilibration of the intracellular pipette solution, the series resistance was kept less than 20 MOhm and then compensated to 8–10 MOhm. The whole-cell pipette solution contained 120 mM CsCl, 10 mM HEPES, 10 mM EGTA, 0.3 mM Na-GTP, 3 mM Mg-ATP and 5 mM QX-314 (pH 7.2, adjusted with CsOH). The bath solution contained 140 mM NaCl, 5 mM KCl, 2 mM MgCl_2_, 2 mM CaCl_2_, 10 mM HEPES-NaOH and 10 mM glucose (pH 7.4). In all the recordings, neurons were voltage clamped at −70 mV. Evoked synaptic responses were recorded with a bipolar electrode placed 100–150 mm from the soma of neurons. Synaptic currents were monitored with an EPC10 amplifier (HEKA). Single extracellular stimulus pulses (90 μA, 1 ms) were controlled with a Model 2100 Isolated Pulse Stimulator (A-M Systems, Inc.) for all evoked EPSCs measurements. EPSCs were pharmacologically isolated by adding the GABAA-receptor blockers picrotoxin (100 μM) to the extracellular solution. Spontaneous miniature excitatory postsynaptic currents (mEPSCs) were monitored in the presence of tetrodotoxin (TTX; 1 μM) to block action potentials. The data were digitized at 10 kHz with a 2-kHz low-pass filter. Miniature events were analyzed in Clampfit 10 (Molecular Devices) using the template matching search and a minimal threshold of 5 pA and each event was visually inspected for inclusion or rejection by an experimenter blind to the recording condition.

### Statistical Analysis

Prism 6.01 (Graphpad) was used for statistical tests, all of which are described in figure legends.

## Results

### Cpx Central α-Helix Itself Is Not Sufficient for Either Clamping Spontaneous Exocytosis or Activation of Ca^2+^ Triggered Exocytosis

**Figure 1:**
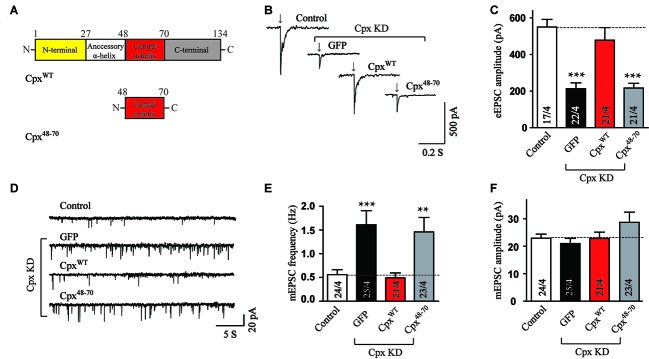
The central α-helix alone cannot regulate neurotransmitter release. **(A)** Schematic structures of wild type (Cpx^WT^) and central α-helix of Cpx (Cpx^48–70^). **(B,C)** Sample traces **(B)** and summary graphs of the amplitude **(C)** of action-potential evoked AMPAR-mediated EPSCs monitored in cultured cortical neurons that were infected with a control lentivirus (control) or lentiviruses expressing complexin shRNAs (GFP) without or with coexpression of Cpx^WT^ or Cpx^48–70^, respectively. **(D–F)** Sample trace **(D)** and summary graphs of the frequency **(E)** and amplitude **(F)** of miniature excitatory postsynaptic currents (mEPSCs) recorded in neurons as described for **(B)**. Data shown in summary graphs are means ± SEM; numbers of cells/independent cultures analyzed are listed in the bars. Statistical assessments were performed by the Student’s *t*-test comparing each condition to the indicated control experiment (**p < 0.01, ***p < 0.001).

To identify the functions of various Cpx structures, we generated Cpx deficient neurons using shRNA dependent KD of Cpx1 and Cpx2 and performed rescue experiments by expressing a series of Cpx1 mutants that were introduced into the same lentivirus used for the KD. Since all the functions of Cpx were relied on the binding of central α-helix to SNARE complex (Maximov et al., 2009), we first examined whether the central α-helix alone could regulate vesicle release. In the action-potential-evoked exocytosis measurement, consistent with previous results, Cpx deficit caused a significant decrease in the amplitude of evoked release. The wild type (Cpx^WT^) but not the central α-helix mutant (Cpx^48–70^) of Cpx1 (Figure 1A) rescued the decrease induced by Cpx1/2 KD (Figures 1B,C), indicating that only the central α-helix couldn’t perform the activated role. To test whether the central α-helix of Cpx1 could clamp spontaneous release, we then measured mEPSCs. As expected, Cpx KD increased the frequency of mEPSCs and the Cpx^WT^ reversed the increase as previous reported. But no obvious suppressing effect in the frequency of mEPSCs was observed in Cpx^48–70^ group (Figures 1D,E), suggesting the central α-helix of Cpx1 was not involved in the clamping function as well. The amplitude of mEPSCs was unchanged in all conditions, suggesting that the effects on the mEPSCs frequency are because of presynaptic processes (Figure 1F). Taking together, our data demonstrated that the central α-helix of Cpx1 alone couldn’t perform any function of Cpx, thus domain combinations were required for Cpx.

### The Accessory and Central α-helices Work Together to Support the Facilitation of Ca^2+^ Triggered Neurotransmitter Release

**Figure 2:**
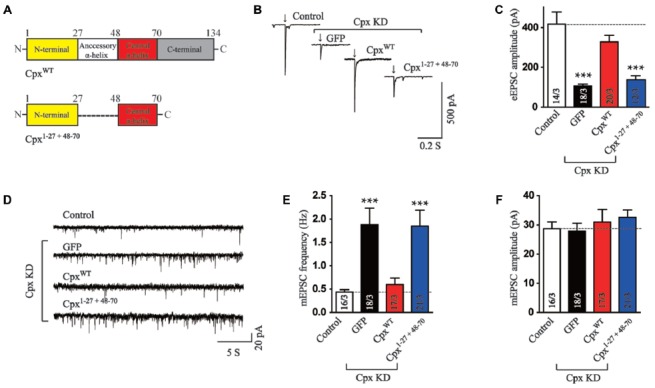
The N-terminal domain is not sufficient to help central α-helix in exocytosis regulation. **(A)** Domain structures of wild type (Cpx^WT^) and the N-terminal domain flanked with central α-helix of Cpx (Cpx^1–27 + 48–70^). **(B,C)** Sample traces **(B)** and summary graphs of the amplitude **(C)** of action-potential evoked AMPAR-mediated EPSCs recorded in cultured cortical neurons that were infected with a control lentivirus (control) or lentiviruses expressing complexin shRNAs (GFP) without or with coexpression of Cpx^WT^ or Cpx^1–27 + 48–70^, respectively. **(D–F)** Sample trace **(D)** and summary graphs of the frequency **(E)** and amplitude **(F)** of mEPSCs monitored in neurons as described for **(B)**. Data shown in summary graphs are means ± SEM; numbers of cells/independent cultures analyzed are listed in the bars. Statistical assessments were performed by the Student’s *t*-test comparing each condition to the indicated control experiment (***p < 0.001).

Besides the central α-helix, Cpx1 has an N-terminal domain, a C-terminal domain and an accessory α-helix. We next want to address whether any of the other three domain could rescue the function of Cpx together with the central α-helix domain. Previous research has clarified that the truncated mutant only containing the central α-helix and C-terminal domain of Cpx1 was not observed any function (Maximov et al., 2009). On the other hand, the N-terminal domain of Cpx1 was reported to trigger vesicle fusion in reconstituted systems (Lai et al., 2016). Therefore, we flanked the N-terminal sequence to central α-helix domain (Cpx^1–27 + 48–70^, Figure 2A) to investigate whether these two domains could rescue any function of Cpx. Surprisingly, different from *in vitro* results, Cpx^1–27 + 48–70^ could not reverse the decrease in excitatory postsynaptic currents (EPSCs) amplitude measurement induced by Cpx KD (Figures 2B,C), indicating only the N-terminal and central α-helix domains were not sufficient to activate exocytosis in neurons. To further analyze the clamp effect of these two domains, the mEPSCs were measured. Our data revealed Cpx^1–27 + 48–70^ had no effect in suppressing the increased frequency of spontaneous release caused by Cpx KD either (Figures 2D–F), thus further suggesting the N-terminal domain was unable to perform the roles of Cpx with the exist of central α-helix domain. The difference between *in vitro* and *in vivo* systems was possible due to more spatial barrier in neurons.

**Figure 3:**
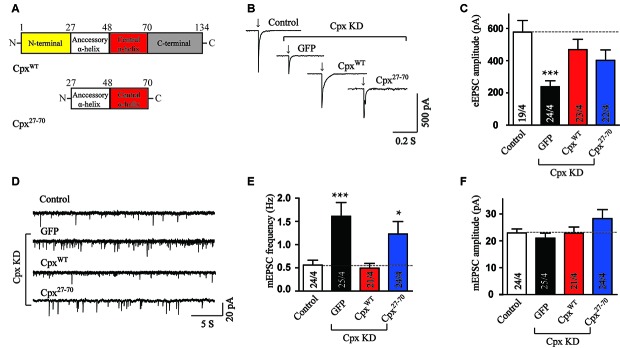
The accessory and central α-helices of complexin selectively activate Ca^2+^ triggered vesicle fusion. **(A)** Domain structures of wild type (Cpx^WT^) and the accessory and central α-helices of Cpx (Cpx^27–70^). **(B,C)** Representative traces **(B)** and summary graphs of the amplitude **(C)** of action-potential evoked AMPAR-mediated EPSCs recorded in cultured cortical neurons that were infected with a control lentivirus (control) or lentiviruses expressing complexin shRNAs (GFP) without or with coexpression of Cpx^WT^ or Cpx^27–70^, respectively. **(D–F)** Sample trace **(D)** and summary graphs of the frequency **(E)** and amplitude **(F)** of mEPSCs monitored in neurons as described for **(B)**. Data shown in summary graphs are means ± SEM; numbers of cells/independent cultures analyzed are listed in the bars. Statistical assessments were performed by the Student’s *t*-test comparing each condition to the indicated control experiment (*p < 0.05, ***p < 0.001).

Since neither the N-terminal nor the C-terminal domain could perform any function of Cpx together with the central α-helix domain, we then wondered whether the accessory α-helix could help the central α-helix domain. For this purpose, the mutant expressed the two α-helices was introduced in Cpx deficient neurons. Interestingly, we found that the accessory and central α-helices (Cpx^27–70^, Figure 3A) expressed together rescued the amplitude of EPSCs (Figures 3B,C), reflecting the activation ability was restored. Moreover, we observed no significant effect in the response time of EPSCs measurements between control and Cpx^27–70^ mutant (Supplementary Figure S1), indicating the synchronization of EPSCs is not altered by Cpx^27–70^. On the contrary, the frequency of mEPSCs was not rescued by expressing the Cpx^27–70^ mutant (Figures 3D–F). Therefore, our results identified that the two α-helices of Cpx1 promoted action-potential triggered neurotransmitter release without the help of N- and C-terminal of Cpx. Consistent with previous results, the clamping role of Cpx was unable to be restored by the accessory and central α-helices also indicated Cpx functions are mechanistically distinct.

### The Vesicular Localization Is Important for Clamping Spontaneous Release

**Figure 4:**
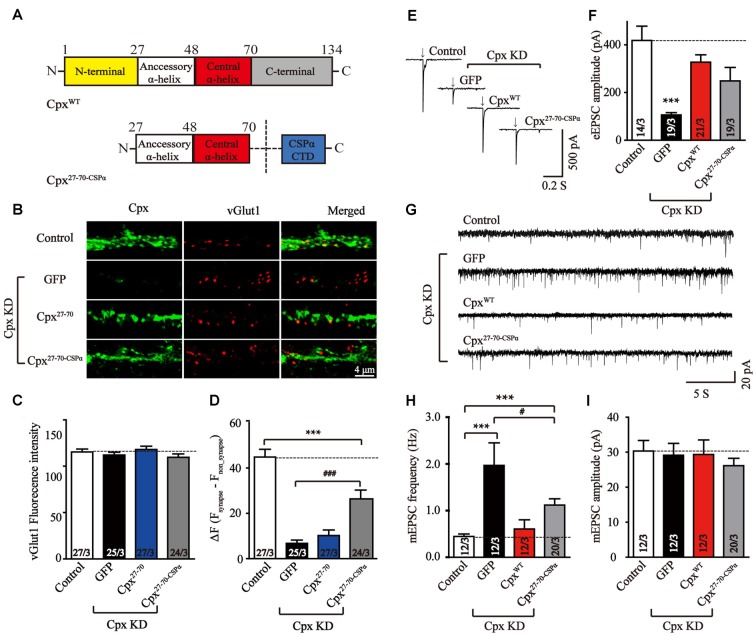
Synaptic vesicle location is important in suppressing spontaneous release. **(A)** Schematic structures of wild type (Cpx^WT^) and the C-terminal sequence of CSPα linked accessory and central α-helices of Cpx (Cpx^27–70-CSPα^). **(B–D)** Representative images **(B)** and summary graphs of vGlut1 intensities **(C)** and ΔF (F_synapse_-F_non-synapse_) of Cpx fluorescence intensities **(D)** of cultured mouse cortical neurons infected with a control lentivirus (control) or lentiviruses expressing complexin shRNAs (GFP) without or with coexpression of Cpx^27–70^ or Cpx^27–70-CSPα^, respectively. **(E,F)** Representative traces **(E)** and summary graphs of the amplitude **(F)** of action-potential evoked AMPAR-mediated EPSCs recorded in cultured cortical neurons that were infected with a control lentivirus (control) or lentiviruses expressing complexin shRNAs (GFP) without or with coexpression of Cpx^WT^ or Cpx^27–70-CSPα^, respectively. **(G–I)** Sample trace **(G)** and summary graphs of the frequency **(H)** and amplitude **(I)** of mEPSCs monitored in neurons as described for **(E)**. Data shown in summary graphs are means ± SEM; numbers of cells/independent cultures analyzed are listed in the bars. Statistical assessments were performed by the Student’s *t*-test comparing each condition to the indicated control experiment (***p < 0.001) or comparing Cpx^27–70-CSPα^ to Cpx knockdown (KD) alone (GFP) experiment (^#^*p* < 0.05, ^###^*p* < 0.001).

We further wanted to uncover the possible reason for the lack of a clamping activity of Cpx^27–70^ in our experiments. Previous studies has demonstrated that lacking the C-terminal domain may cause mislocation of Cpx1 and result the increase of mEPSCs frequency (Gong et al., 2016). Targeting Cpx lacking C-terminal sequence mutant to vesicle membrane by fusing the C-terminal palmitoylated sequence of CSPα (Zhou et al., 2013) suppressed spontaneous mini release. To test whether synaptic vesicle localization could rescue the clamping activity of Cpx^27–70^, we designed a chimera that fused CSPα at the end of Cpx^27–70^ (Cpx^27–70-CSPα^, Figure 4A). To test whether the C-terminal sequence of CSPα could really drive the two α-helices of Cpx1 to vesicle membrane, immunocytochemistry experiments were performed in lentivirus-infected neurons. Consistent with previous results, comparing to the Cpx deficit neurons, the synaptic located Cpx^27–70-CSPα^ but not Cpx^27–70^ significant increased (Figures 4B–D), confirming the vesicle located ability of CSPα. However, the synaptic signal of Cpx^27–70-CSPα^ was lower than control neurons, suggesting Cpx^27–70-CSPα^ only partially rescued the vesicle localization of Cpx1. We then found that Cpx^27–70-CSPα^ rescued the decrease of EPSCs amplitude induced by Cpx deficit, however the efficiency is slight but not significant lower than that of Cpx^WT^ (Figures 4E,F), confirming the two α-helices are able to activate Ca^2+^ triggered fast synchronous exocytosis. Again, Cpx^27–70-CSPα^ doesn’t affect the synchronization of EPSCs by reflecting as an unaltered response time (Supplementary Figure S2). Moreover, comparing to the neurons lacking Cpxs, the increase of mEPSCs frequency was partially but significantly reversed by Cpx^27–70-CSPα^ expression (Figures 4G–I), consistent with the partially rescued localization caused by Cpx^27–70-CSPα^, confirming synaptic vesicle localization had positive role in clamping spontaneous vesicle fusion for Cpx1. However, different from the mutant only lacking C-terminal domain but fused with CSPα (Gong et al., 2016), the mini frequency in Cpx^27–70-CSPα^ expressed neurons was still higher than control neurons (Figures 4G–I), thus arguing the N-terminal sequence might have a role in clamping effect of Cpx1 as well.

## Discussion

Through binding to SNARE complex, Cpxs perform at least two physiological functions: activating fast synchronous release and clamping spontaneous vesicle fusion (Yang et al., 2010; Gong et al., 2016; Trimbuch and Rosenmund, 2016). These functions of Cpx selectively depend on distinct modular sequences (Xue et al., 2007; Yang et al., 2010, 2015). The conserved activation function of Cpx is found across all species and different types of Ca^2+^ triggered synaptic neurotransmitter release (Reim et al., 2001; Maximov et al., 2009; Kaeser-Woo et al., 2012; Cao et al., 2013; Yang et al., 2013), thus considering as the key role of Cpx. While clamping spontaneous release by Cpx is less conserved among species and varies depending on experimental conditions. Knockout Cpx in mice autaptic neurons claims no obvious clamping effect (Reim et al., 2001; Xue et al., 2007). In contrast, KD Cpx in high density mouse neuronal cultures, as well as knockout Cpx in *Drosophila* and *C. elegans*, increases spontaneous release (Huntwork and Littleton, 2007; Yang et al., 2010; Martin et al., 2011; Jorquera et al., 2012; Kaeser-Woo et al., 2012), supporting the fusion clamp model.

In the present study, we made the following observations. First, expressing the central α-helices of Cpx alone is not sufficient to support its function. Second, the accessory α-helix but not the N-terminal domain of Cpx together with central α-helix plays a role in activation of Ca^2+^ triggered synchronous release but not in clamping spontaneous release. Third, targeting the two α-helices of Cpx to synaptic vesicle suppresses the spontaneous vesicle fusion, but the role of N-terminal sequence cannot be ignored in clamping.

Our results extend some of the previous studies, for example, confirming the role of C-terminal domain of Cpx in clamping via its synaptic vesicle localization ability and reemphasizing the distinct structural dependent functions of Cpx. But our data are inconsistent with some researches as well. The main difference is how to understand the role of N-terminal domain. Lacking N-terminal domain of Cpx in culture neurons was reported an inactivation in Ca^2+^ triggered release (Maximov et al., 2009), and expressing N-terminal domain of Cpx independently activate Ca^2+^ triggering fusion in reconstituted single-vesicle fusion assay (Lai et al., 2016). These observations strongly argued an activation function for N-terminal sequence. Previous studies also identified that the N- and C-terminal domain interacted with plasma membrane (Lai et al., 2016) and vesicle membrane (Gong et al., 2016), respectively. Therefore a hypothesis is led that the N- and C-terminal domain keep a balance position for Cpx via their trans-membrane interaction. Lacking N-terminal domain kept Cpx away from plasma membrane, thus abolished the activation ability. While Lacking C-terminal domain pushed Cpx more close to plasma membrane to impair the clamping effect. The absence of both N- and C-terminal domain lost all membrane interaction, thus free the two α-helices to activate vesicle fusion. The activation was slightly decreased when CSPα sequence drove the two α-helices more close to vesicle membrane also supported this hypothesis. Moreover, flanked expressing the N-terminal domain and central α-helix may not simultaneously bind to SNARE complex and plasma membrane in culture neurons, thus not activate release.

In conclusion, we here reveal the general activation role of Cpx via its accessory and central α-helices, and argue the synergistic effect of N-terminal sequence and synaptic vesicular localization by C-terminal domain in clamping spontaneous synaptic vesicle exocytosis.

## Author Contributions

YY and SC carried out the experiments. YY, SC, XM, JG and CL analyzed the data. XY contributed to the planning of the work and wrote the article.

## Conflict of Interest Statement

The authors declare that the research was conducted in the absence of any commercial or financial relationships that could be construed as a potential conflict of interest.
