# Supplementary figures and images for: Accessory and Central α-helices of Complexin Selectively Activate Ca2+ Triggering of Synaptic Exocytosis
Source: Front Mol Neurosci. 2018 Feb 26;11:61. doi: 10.3389/fnmol.2018.00061 (PMC5834437; doi:10.3389/fnmol.2018.00061)

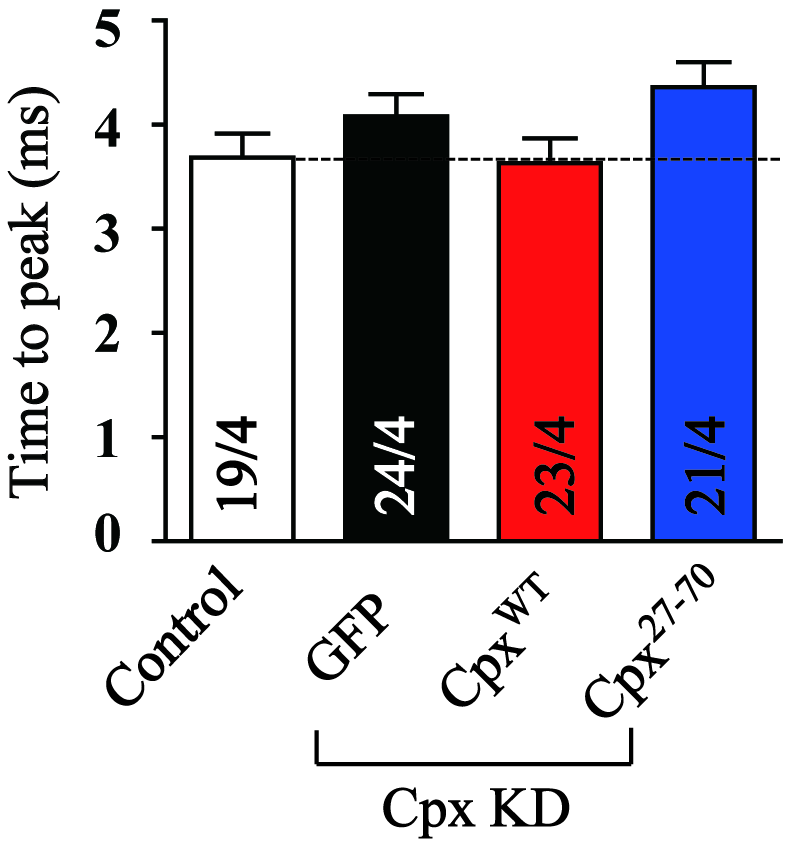

Supplement: Supplementary file 1 [file Image_1.tif]

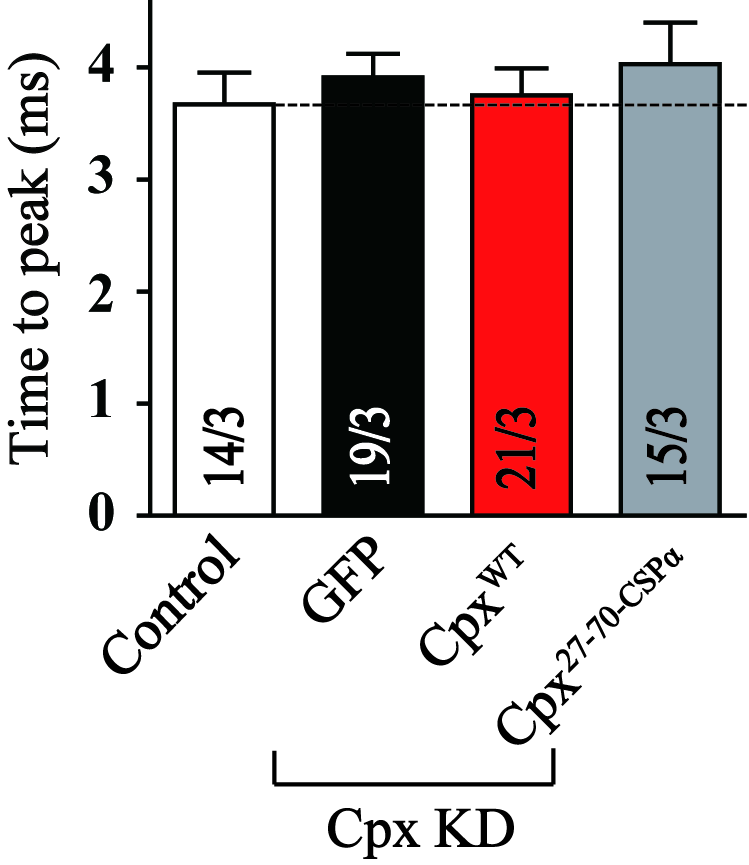

Supplement: Supplementary file 2 [file Image_2.tif]
